# Identification of candidate genes regulating seed oil content by QTL mapping and transcriptome sequencing in *Brassica napus*

**DOI:** 10.3389/fpls.2022.1067121

**Published:** 2022-12-08

**Authors:** Zhongchun Xiao, Chao Zhang, Cunmin Qu, Lijuan Wei, Liyuan Zhang, Bo Yang, Kun Lu, Jiana Li

**Affiliations:** ^1^ Key Laboratory of Biological Genetic Resources Mining and Molecular Breeding of Qianxinan Prefecture, College of Biology and Chemistry, Minzu Normal University of Xingyi, Xingyi, China; ^2^ Guizhou Oil Research Institute, Guizhou Academy of Agricultural Sciences, Guiyang, China; ^3^ Academy of Agricultural Sciences, Southwest University, Chongqing, China

**Keywords:** *Brassica napus*, seed oil content, QTL mapping, differentially expressed genes (DEGs), candidate genes

## Abstract

Increasing oil production is a major goal in rapeseed (*Brassica napus*) molecular breeding programs. Identifying seed oil content (SOC)-related candidate genes is an important step towards achieving this goal. We performed quantitative trait locus (QTL) mapping of SOC in *B. napus* using a high-density SNP genetic map constructed from recombinant inbred lines and the Illumina Infinium^TM^ 60K SNP array. A total of 26 QTLs were detected in three years on A01, A03, A05, A06, A09, C01, C03 and C05, which accounted for 3.69%~18.47% of the phenotypic variation in SOC. Of these, 13 QTLs are reported here for the first time. 1713 candidate genes in the 26 QTLs confidence interval were obtained. We then identified differentially expressed genes (DEGs) between the high- and low-SOC accessions, to narrow down our focus to 21 candidate genes (Y1-Y21) related to SOC, and we will focus on 11 (Y1-Y11) candidate genes that contribute to the formation of high-SOC. In addition to providing insight into the genetic basis of SOC in *B. napus*, the loci identified and candidate genes in this study can be used in molecular breeding strategies to increase SOC in this important seed crop.

## Introduction

Due to its low saturated fatty acid content, rapeseed (*Brassica napus*) oil is considered a healthy edible vegetable oil. Increasing oil production, by increasing seed yield and seed oil content (SOC), is a major goal of *B. napus* breeding. Tremendous progress has been made towards increasing *B. napus* oil production, especially in China, due to the development of next-generation sequencing technology and SNP markers. *B. napus* breeders in China have produced germplasm with a SOC of 55–60%, with the potential to increase it to 75% (Hu et al., 2013; Hua et al., 2016). Therefore, there is a broad prospect for improving the SOC of *B. napus* in China.

The SOC of *B. napus* is a complex quantitative trait, controlled by multiple genes and environmental effects (Si et al., 2003; Liu et al., 2016). Previous studies have shown that the SOC of *B. napus* is mainly determined by the maternal genotype, and the photosynthesis of maternal silique pericarps plays an important role in regulating SOC (Hua et al., 2012; Wang et al., 2010). As for the QTL mapping of SOC in *B. napus*, the previous studies have mapped many related QTLs. However, due to differences in the subsets of accessions used, genetic maps, localization methods, and environmental differences in the populations used for QTL mapping, the QTLs associated with SOC in *B. napus* obtained by predecessors are also very different. Out of 14 and 10 SOC-related QTLs discovered in the DY and RNSL DH populations, respectively, only one was detected simultaneously in both populations (Delourme et al., 2006). Yan et al. (2009) detected 11 QTLs related to SOC in three different environments using the *B. napus* recombinant inbred line (RIL) population. These QTLs were mainly distributed on C05 and C06 chromosome, with a single QTL accounting for 5.19-13.57% of the phenotypic variation. Chen et al. (2010) detected 27 SOC-related QTLs distributed in 14 linkage groups under nine different environments, using a *B. napus* DH population, of which a single QTL explained 4.2-30.2% of the phenotypic variation (Chen et al., 2010). Zhao et al. (2012) reconstructed a map using the SG-DH population and detected nine SOC-related QTLs, located on chromosomes A01, A05, A07, A09, C02, C03, C06 and C08, under 11 different growth regimes, which together accounted for 57.79% of the phenotypic variation (Zhao et al., 2012). Wang et al. (2013) constructed a *B. napus* KNDH population using “KenC-8” and “N53-2” and identified 24 SOC-related QTLs (Wang et al., 2013). Sun et al. (2016) constructed two F_2_ populations and obtained 40 SOC-related QTLs using linkage mapping under different environments (Sun et al., 2016).

In this study, we aimed to identify candidate genes associated with SOC by combining QTL mapping and transcriptome sequencing between extremely high- and low-SOC *B. napus* accessions. We created a high-generation RIL containing 186 strains, and constructed a high-density genetic linkage map, consisting of 8,575 SNP markers and 1,201 clusters across the 6140.2cM *B. napus* genome, using the 60K SNP chip. Finally, a total of 26 SOC-related QTLs were detected. Meanwhile, based on differential expression genes (DEGs) between the high- and low-SOC accessions from our previous study (Xiao et al., 2019), we identified 21 candidate genes, which could be useful for further gene cloning and molecular breeding for higher SOC in *B. napus*.

## Materials and methods

### Plant materials and phenotyping

The high-seed oil content (SOC; 44.57%) *B. napus* accession, GH06, was used as the female parent and a low-SOC (36.69%) accession, ZY821, as the male parent. Starting from the F_2_ generation, plants were self-fertilized using the one grain transfer method. In the 10th generation, a high-generation recombinant inbred line (RIL) population containing 186 strains was constructed and cultivated in the rapeseed planting base of Xiema Town, Beibei District, Chongqing (29°45′39.99” N, 106°22′38.47”E, 238.57 m), for three consecutive years (2016-2018). All field experiments followed a randomized complete block design with two replicates. From each accession, 30 plants were grown in three rows per plot, with 10 plants per row, with a row spacing of 40 cm, and a plant spacing of 20 cm. The trial was managed in a conventional manner to ensure a consistent growth environment for all accessions. At maturity, five representative plants were collected from the middle of each plot. The oil content of the desiccated seeds was measured by near-infrared reflectance spectroscopy (NIRS DS2500).

### Statistical analysis

The SOC data from three consecutive years were compared using a Student’s *t*-test in Microsoft Excel 2013, and the normal distribution map was generated using Origin Pro 8.0. The correlation analysis of the RIL population was performed using DPS7.05 statistical analysis software. Best linear unbiased prediction (BLUP) for SOC was evaluated using an R script (http://www.eXtension.org/pages/61006). The coefficient of variation was calculated using the formula CV = σ/μ, where σ is the standard deviation and μ is the average.

### Genetic map construction

Five young leaves of each accession were mixed, and DNA was extracted for SNP marker analysis. DNA samples were pretreated, chip hybridized, eluted, single base extended, stained, and embedded according to Illumina’s Infinium^TM^ HD Assay Ultra instructions. The chip was scanned using Illumina HiSCAN, and the results were analyzed using GenomeStudio genotyping software v2011, to obtain the genotype of each accession. The SNP genetic map was constructed using MSTmap software (Wu et al., 2008), and all markers were grouped using a minimum threshold LOD score of 5.0. The order of markers on each linkage group was calculated using the minimum recombination frequency between the markers. The SNP genetic map included 8,575 SNP markers and 1,201 bins, and covered 6340.2 cM of the *B. napus* genome.

### QTL mapping

The QTL mapping of SOC was performed in WinQTLCart 2.5 software (Silva Lda et al., 2012) using the composite interval mapping (CIM) method. The LOD threshold was set to 2.0. Each QTL was named after the year and the italic lowercase “*q*”, followed by the trait name, chromosome, and QTL serial number. For example, *2016-qOCA05-1* indicates the first QTL for SOC on chromosome A05 in 2016.

### Differentially expressed genesin high- and low-SOC accessions

One low-SOC (CQ46: Ningyou12, 33.0% of SOC) and two high-SOC (CQ24: SWU47, 42.7% of SOC and CQ52: Zhongshuang11, 43.3% of SOC) lines were selected for transcriptome sequencing (RNA Seq), and DEGs were obtained in seeds 30 days after flowering on the main inflorescence (30SM) and on the primary branch (30SB) based on our previous research (Xiao et al., 2019), and the RNA-Seq datasets presented in this study can be found in online repositories. The names of the repository/repositories and accession number(s) can be found below: BIG Data Center under BioProject accession number PRJNA602979.

### Screening of SOC−related candidate genes

To screen for candidate genes related to SOC, we combined all candidate genes in the QTL confidence interval, with DEGs between extremely high- and low- SOC accessions in 30SM and 30SB. Finally, candidate genes that are up-regulated and down-regulated in both 30SM and 30SB of high-SOC rapeseed (CQ24 and CQ52) are identified to be important candidate genes related to SOC, and candidate genes that contribute to the formation of high-SOC will be focused on in this study.

### Quantitative real−time polymerase chain reaction analysis

Total cDNA was synthesized from 1,000 ng RNA according to the manufacturer’s instructions (Perfect Real Time; TaKaRa Biotechnology, Dalian, China). qRT-PCR Primers are listed in **Table S3**. Each reaction contained 10 μL TBGreen II (TakaRa), 2.0 μL cDNA, 1.6 μL primer, 0.4 μL ROX Reference Dye II, and distilled water to a final volume of 20 μL. The PCR program was as follows: 95 °C for 30 s and 35 cycles of 95 °C for 5 s, followed by 56-62 °C (depending on the primers used) for 30 s. Each reaction including three biological replicates, relative expression levels were obtained using the 2^−ΔΔCt^ method, and *BnACTIN7* was used as internal control.

## Results

### Phenotypic variation of SOC

**Figure 1:**
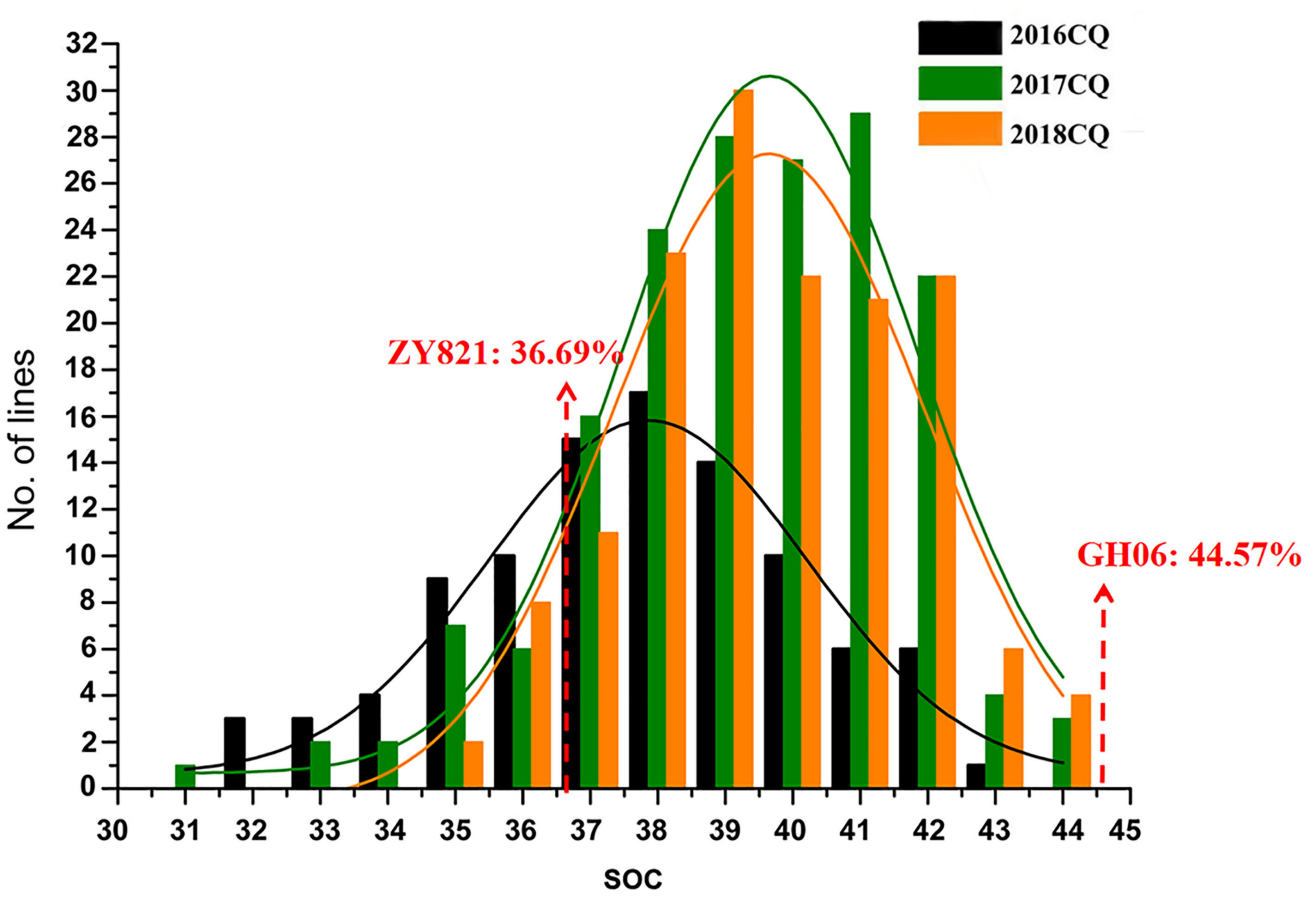
Frequency distribution of seed oil content (SOC, % of seed weight) of RIL populations in different environments. “CQ “refers to Chongqing and “No. of Lines” represents the number of accessions.

In current study, extensive phenotypic variations of seed oil content (SOC) were found (**Table 1**), and seed oil content (SOC, % of seed weight) phenotypes in three environments (2016CQ-2018CQ) of 186 lines for QTL mapping are shown in **Table S1**. SOC ranged from 31.32% to 42.6%, with an average of 37.2%, in 2016CQ, from 30.97% to 43.87%, with an average of 38.81%, in 2017CQ, and from 34.18% to 43.54%, with an average of 39.11%, in 2018CQ. The coefficient of variation (CV) was 6.59%, 5.95% and 5.09% in 2016CQ, 2017CQ, and 2018CQ, respectively. This indicated that the variation of SOC in the three environments is small and stable. The SOC varied continuously in the three environments and approached a normal distribution (**Figure 1**), indicating that this trait is controlled by multiple genes.

**Table 1:** Phenotypic variation in seed oil content (SOC) in the recombinant inbred line (RIL) population.

Trait	Env.	Mean ±SD(%)	Min (%)	Max (%)	CV(%)
SOC	2016CQ	37.2±2.45	31.32	42.6	6.59
	2017CQ	38.81±2.31	30.97	43.87	5.95
	2018CQ	39.11±1.99	34.18	43.54	5.09

CQ, Chongqing; SD, standard deviation; CV, coefficient of variation.

### Correlation analysis between SOC and other traits

The phenotypic correlation analysis between SOC and 12 other traits indicated (**Table 2**) that there was positive correlation between SOC and oil + protein content, erucic acid, glucosinolate, linolenic acid, economic yield, biological yield, and harvest index. Besides, there was negative correlation between SOC and protein content, linoleic acid, stearic acid, oleic acid, and palmitic acid. And the significance of the correlation between SOC and other traits was shown in **Table 2**. Therefore, increasing the SOC has an extremely important role in production practice.

**Table 2:** Correlation coefficients of seed oil content (SOC) with 12 other traits in a *B. napus* RIL population grown in different environments.

Trait	Env.	Protein content	Oil+Protein content	Erucic acid	Glucosinolate	Linolenic acid	Linoleic acid	Stearic acid	Oleic acid	Palmitic acid	Economic yield	Biological yield	Harvest index
SOC	2016CQ	-0.270**	0.857**	0.475**	0.148	0.412**	-0.639**	-0.442**	-0.406**	-0.730**	0.331**	0.221*	0.334**
	2017CQ	-0.346**	0.853**	0.505**	0.181*	0.058	-0.622**	-0.449**	-0.436**	-0.754**	0.409**	0.116	0.554**
	2018CQ	-0.565**	0.762**	0.200*	0.065	0.156*	-0.352**	-0.346**	-0.099	-0.454**	0.064	0.004	0.149

* and ** indicate significant differences at p < 0.05 and p < 0.01, respectively.

### QTL mapping for SOC

**Figure 2:**
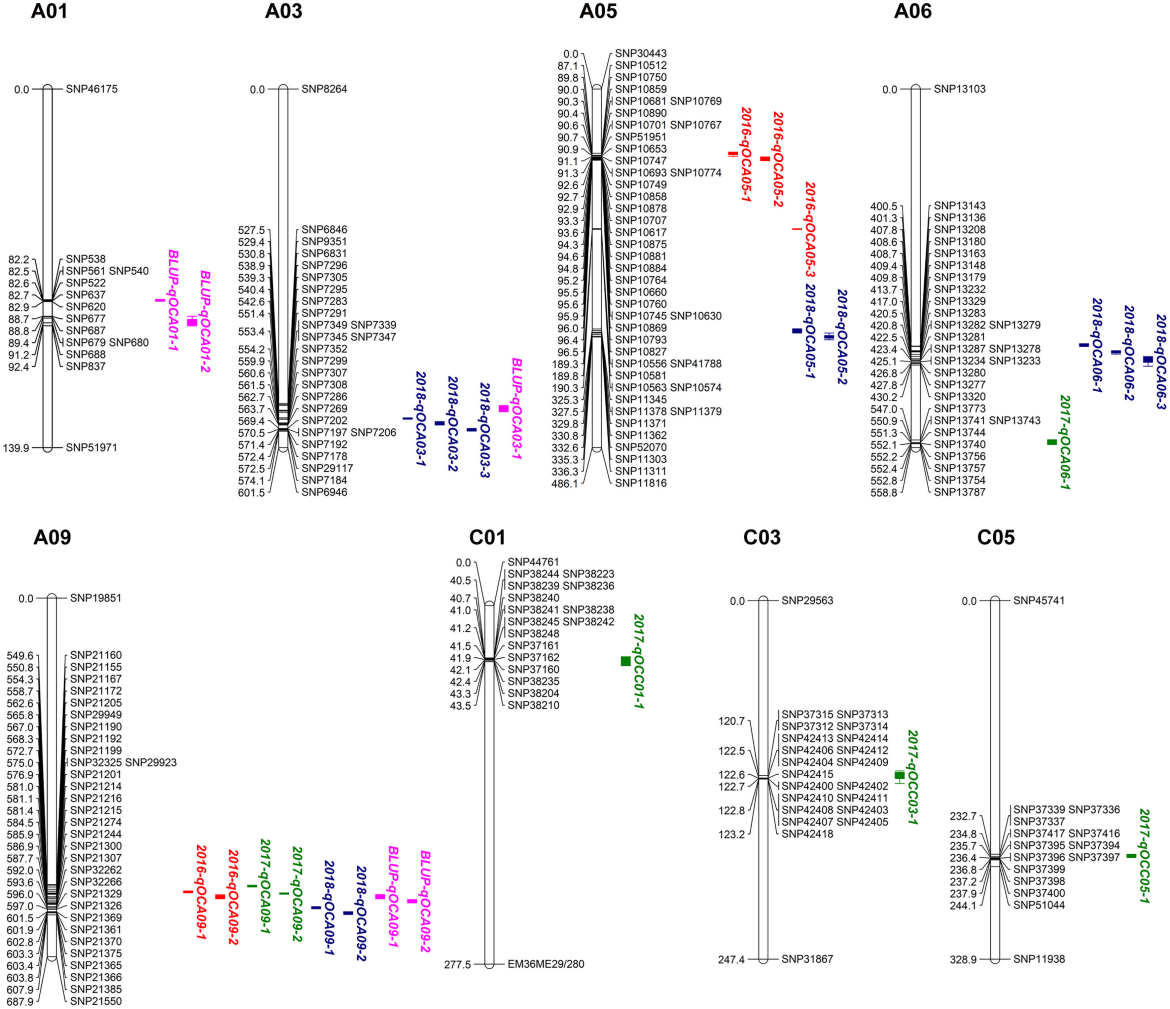
Putative QTL locations of seed oil content on the SNP genetic map. Red, green, blue and pink fonts represent QTL detected in 2016CQ, 2017CQ, 2018CQ and BLUP, respectively.

QTL mapping for SOC was performed using the composite interval mapping (CIM) method. When the LOD value was≥2.0, we detected 26 SOC-related QTLs in three environments, located on chromosomes A01, A03, A05, A06, A09, C01, C03 and C05 (**Table 3**, **Figure 2**). These QTLs explained 3.69-18.47% of the phenotypic variation (R^2^). The QTL *2017-qOCA09-2*, located at 30.08-30.31 Mb on chromosome A09, explained the highest proportion of the phenotypic variation (18.47%). We also identified several overlapping QTLs: *2016-qOCA09-1* and *2017-qOCA09-2*; *2016-qOCA09-2* and *BLUP-qOCA09-1*; *2018-qOCA09-1* and *BLUP-qOCA09-2*; *2016-qOCA05-1* and *2016-qOCA05-2*; *2018-qOCA03-1* and *2018-qOCA03-2*; and *2018-qOCA05-1* and *2018-qOCA05-2*. The additive effects of these overlapping QTLs are positive, indicating that the synergistic genes affecting SOC are mainly derived from the female parent (GH06), which is consistent with maternal control of SOC in *B. napus* (Hua et al., 2012).

**Table 3:** QTL for seed oil content (SOC) detected from the RIL population in three environments.

QTL name	Env.	Chr.	LOD Score	SNP interval	Additive effect	R2(%)	Physical interval (bp)	Candidate genes in confidence interval	Detected in previous studies
2016-*q*OCA05-1	2016CQ	ChrA05	3.71	SNP10512-SNP10774	1.43	11.10	9891125/13621300	BnaA05g15440D-BnaA05g18300D	
2016*-q*OCA05-2	2016CQ	ChrA05	2.11	SNP10749-SNP10827	1.23	6.56	10943170/13562855	BnaA05g16290D-BnaA05g18300D	
2016*-q*OCA05-3	2016CQ	ChrA05	2.96	SNP10556-SNP10574	-1.57	9.52	10799861/10877060	BnaA05g16210D-BnaA05g16250D	
2016-*q*OCA09-1	2016CQ	ChrA09	2.36	SNP21172-SNP21190	0.69	7.37	30078610/30361587	BnaA09g43400D-BnaA09g44150D	qOC-A9-4-TN (Jiang et al., 2014)
2016-*q*OCA09-2	2016CQ	ChrA09	4.52	SNP21199-SNP29923	0.93	13.44	30500499/30521739	BnaA09g44410D-BnaA09g44450D	cqOC-A9-9 (Chao et al., 2017); qOC-A9-4-TN (Jiang et al., 2014)
2017*-q*OCA06-1	2017CQ	ChrA06	2.62	SNP13773-SNP13754	0.52	4.91	20956439/21140416	BnaA06g31190D-BnaA06g31470D	
2017-*q*OCA09-1	2017CQ	ChrA09	4.66	SNP21160-SNP21167	0.79	10.81	29917465/30024926	BnaA09g43040D-BnaA09g43270D	qOC-A9-4-TN (Jiang et al., 2014)
2017-*q*OCA09-2	2017CQ	ChrA09	9.03	SNP21172-SNP21192	1.02	18.47	30078610/30386495	BnaA09g43400D-BnaA09g44200D	qOC-A9-4-TN (Jiang et al., 2014); Bn-A09-p32713083 (Liu et al., 2016)
2017-*q*OCC01-1	2017CQ	ChrC01	2.20	SNP38244-SNP38210	-0.47	3.94	11159198/11021503	BnaC01g15970D-BnaC01g16200D	Bn-scaff_17592_1-p654560 (Liu et al., 2016)
2017-*q*OCC03-1	2017CQ	ChrC03	2.82	SNP37315-SNP42418	0.52	4.73	47596910/46916297	BnaC03g57710D-BnaC03g58260D	
2017-*q*OCC05-1	2017CQ	ChrC05	2.23	SNP37339-SNP51044	0.47	3.97	40215021/40656747	BnaC05g43600D-BnaC05g44170D	cqOC-C5-7, cqOC-C5-8 (Chao et al., 2017)
2018*-q*OCA03-1	2018CQ	ChrA03	3.19	SNP7291-SNP7352	0.54	6.70	27827670/28701263	BnaA03g53180D-BnaA03g54210D	
2018*-q*OCA03-2	2018CQ	ChrA03	3.20	SNP7269-SNP7299	0.54	6.39	27627877/27900254	BnaA03g52920D-BnaA03g53200D	
2018*-q*OCA03-3	2018CQ	ChrA03	2.42	SNP7184-SNP7202	0.49	4.93	27277899/27337191	BnaA03g52340D-BnaA03g52400D	
2018*-q*OCA05-1	2018CQ	ChrA05	2.74	SNP11345-SNP11362	0.50	5.60	17522874/17737620	BnaA05g23110D-BnaA05g23420D	
2018*-q*OCA05-2	2018CQ	ChrA05	4.04	SNP11311-SNP11362	0.60	8.13	17277976/17737620	BnaA05g22780D-BnaA05g23420D	snp842906 (Wang et al., 2018)
2018*-q*OCA06-1	2018CQ	ChrA06	4.70	SNP13136-SNP13143	0.64	9.61	17149710/17214356	BnaA06g24680D-BnaA06g24800D	
2018*-q*OCA06-2	2018CQ	ChrA06	4.91	SNP13208-SNP13232	0.66	10.00	17811068/18056356	BnaA06g25740D-BnaA06g26190D	RNSL-qOC-A6 (Delourme et al., 2006); Bn-A06-p16689717 (Liu et al., 2016)
2018*-q*OCA06-3	2018CQ	ChrA06	2.43	SNP13320-SNP13329	0.47	5.19	18702433/18809438	BnaA06g27220D-BnaA06g27410D	
2018*-q*OCA09-1	2018CQ	ChrA09	4.80	SNP21215-SNP21326	0.68	10.45	30687778/31045659	BnaA09g44730D-BnaA09g45440D	cqOC-A9-9 (Chao et al., 2017); qOC-A9-4-TN (Jiang et al., 2014)
2018*-q*OCA09-2	2018CQ	ChrA09	4.22	SNP21369-SNP21385	0.61	8.61	31297302/31720463	BnaA09g45920D-BnaA09g46870D	cqOC-A9-10 (Chao et al., 2017)
BLUP*-q*OCA01-1	BLUP	ChrA01	4.51	SNP538-SNP620	-0.36	7.34	14133580/14587546	BnaA01g21840D-BnaA01g22210D	
BLUP*-q*OCA01-2	BLUP	ChrA01	5.40	SNP677-SNP837	-0.38	8.73	15577785/15683250	BnaA01g23190D-BnaA01g23330D	
BLUP*-q*OCA03-1	BLUP	ChrA03	2.53	SNP6846-SNP7283	0.25	3.69	24438882/27800984	BnaA03g47620D-BnaA03g53140D	*TN-qOC-A3-1* (Jiang et al., 2014); Bn-A03-p2948394 (Liu et al., 2016)
BLUP*-q*OCA09-1	BLUP	ChrA09	8.96	SNP21192-SNP21214	0.51	15.93	30386495/30681504	BnaA09g44210D-BnaA09g44720D	cqOC-A9-9 (Chao et al., 2017); qOC-A9-4-TN (Jiang et al., 2014); Bn-A09-p32713083,Bn-A09-p32864411 (Liu et al., 2016)
BLUP*-q*OCA09-2	BLUP	ChrA09	9.76	SNP21214-SNP21326	0.53	17.12	30681504/31045659	BnaA09g44720D-BnaA09g45440D	cqOC-A9-9 (Chao et al., 2017); qOC-A9-4-TN (Jiang et al., 2014)

### Screening of SOC−related candidate genes

We identified a total of 1,713 candidate genes within the mapping interval of the 26 SOC-related QTLs (**Table S2**). These were compared with the genes that were differentially expressed in 30SM and 30SB between extremely high- and low- oil content accessions. Finally, 11 genes that were up-regulated in both high- oil content (CQ24 and CQ52) accessions compared to low- oil content (CQ46) accession were screened as important candidate genes (Y1-Y11) that contribute to the formation of high SOC, and 10 genes that were down-regulated in both high- oil content (CQ24 and CQ52) accessions compared to low- oil content (CQ46) accession (Y12-Y21) were also considered candidate genes that affect SOC, and the details are listed in **Table 4**.

**Table 4:** Identification of candidate genes by combining QTL mapping with RNA-Seq.

Code	Candidate genes	Physical position	*Arabidopsis*homologue	Functional description
Y1	BnaA01g22130D	ChrA01:14483566-14484250	Unknown	Unknown
Y2	BnaA03g48490D	ChrA03:24882893-24883369	AT4G27160	Seed storage albumin 3 (SESA3)
Y3	BnaA03g48800D	ChrA03:25043346-25045679	AT4G27760	FOREVER YOUNG (FEY)
Y4	BnaA03g50010D	ChrA03:25916589-25917524	AT4G30220	Small nuclear ribonucleoprotein F (RUXF)
Y5	BnaA03g50730D	ChrA03:26337386-26338898	AT3G48000	Aldehyde dehydrogenase 2B4 (ALDH2B4)
Y6	BnaA03g52350D	ChrA03:27303002-27304714	Unknown	Unknown
Y7	BnaA03g52640D	ChrA03:27453028-27455603	AT1G61180	LRR and NB-ARC domains-containing disease resistance protein
Y8	BnaA03g52660D	ChrA03:27458057-27461975	AT4G33630	EXECUTER1 (EX1)
Y9	BnaA03g53510D	ChrA03:28094719-28096583	AT4G36650	Plant-specific TFIIB-related protein (PBRP)
Y10	BnaA03g53860D	ChrA03:28425197-28427930	Unknown	Unknown
Y11	BnaA09g43150D	ChrA09:29959554-29960484	AT2G21490	Dehydrin LEA (LEA)
Y12	BnaA03g48780D	ChrA03:25039515-25040991	AT4G27680	P-loop containing nucleoside triphosphate hydrolases superfamily protein
Y13	BnaA03g49200D	ChrA03:25322382-25322744	AT4G28365	Early nodulin-like protein 3 (ENODL3)
Y14	BnaA03g49250D	ChrA03:25378962-25382374	AT4G28410	Tyrosine transaminase family protein
Y15	BnaA03g52310D	ChrA03:27267291-27268611	AT4G33000	Calcineurin B-like protein 10 (CBL10)
Y16	BnaA06g31450D	ChrA06:21135320-21135661	AT3G28500	60S acidic ribosomal protein family
Y17	BnaA09g43660D	ChrA09:30169090-30169437	AT2G20619	Plant thionin family protein
Y18	BnaA09g45090D	ChrA09:30910078-30910815	AT1G17180	Glutathione S-transferase TAU 25 (GSTU25)
Y19	BnaA09g45320D	ChrA09:30987973-30988607	AT1G14980	Chaperonin 10 (CPN10)
Y20	BnaA09g46300D	ChrA09:31492745-31493868	AT2G37550	ARF-GAP domain 7 (AGD7)
Y21	BnaC05g43660D	ChrC05:40410175-40411373	AT3G08900	Reversibly glycosylated polypeptide 3 (RGP3)

### Validation of candidate genes by qRT−PCR analysis

**Figure 3:**
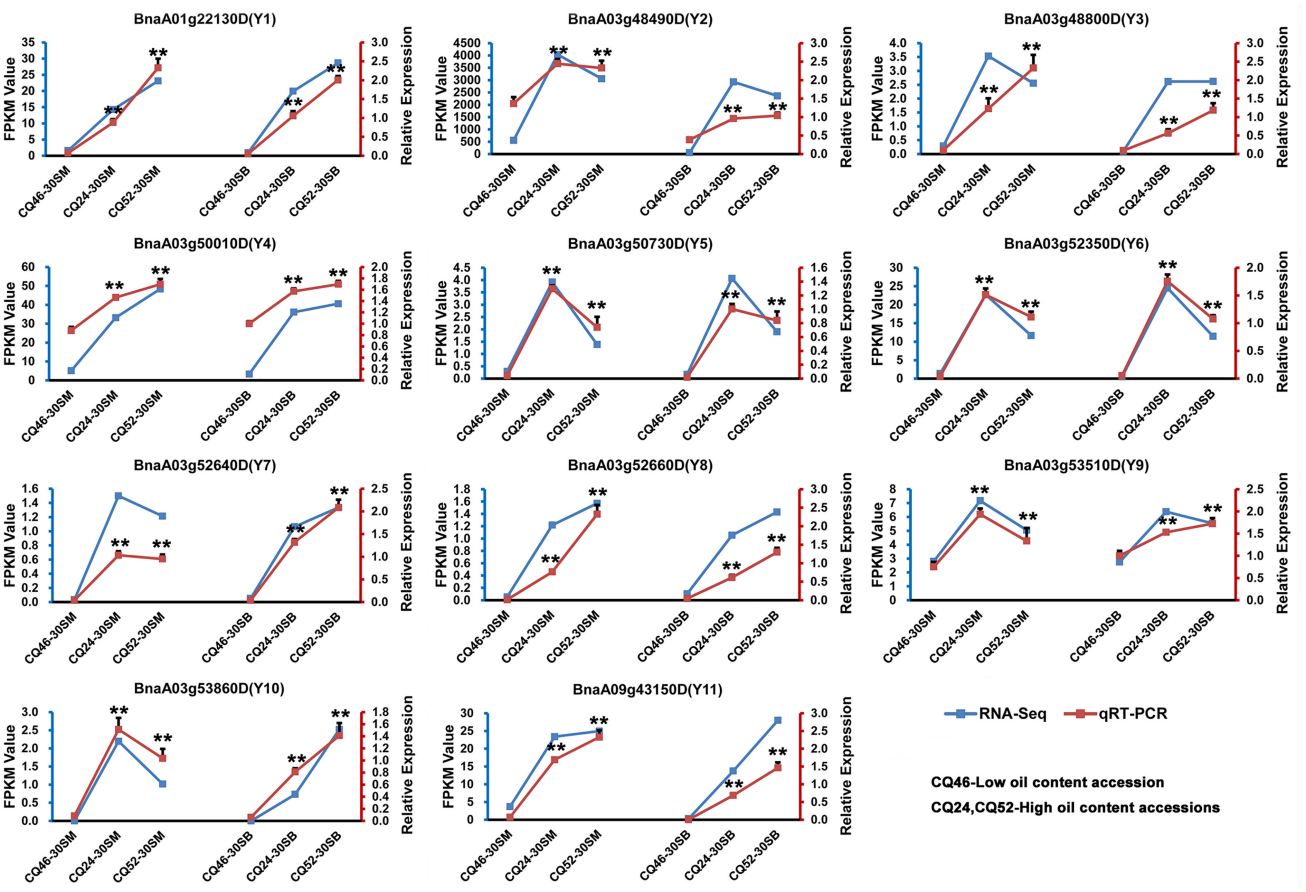
qRT-PCR analysis confirmed the accuracy of transcriptome sequencing by validating the expression patterns of 11 candidate genes in seeds 30 days after flowering on the main inflorescence (30SM) and on the primary branch (30SB). The blue line represents the RNA-Seq results and the red line represents the qRT-PCR results. **Denotes significance differences with P < 0.01, based on Student’s *t*-test.

To confirm the accuracy of the RNA-Seq results, 11 candidate genes (Y1-Y11) were performed qRT-PCR analysis, and the expression levels of these genes in 30SM and 30SB between high-SOC (CQ24 and CQ52) and low-SOC accessions (CQ46) by qRT-PCR and transcriptome sequencing (RNA-Seq) are shown in **Figure 3**. And the qRT-PCR results showed highly consistent with RNA-Seq, which fully demonstrated the reliability and accuracy of the RNA-Seq data.

### Expression patterns of candidate genes in seeds at different developmental stages

**Figure 4:**
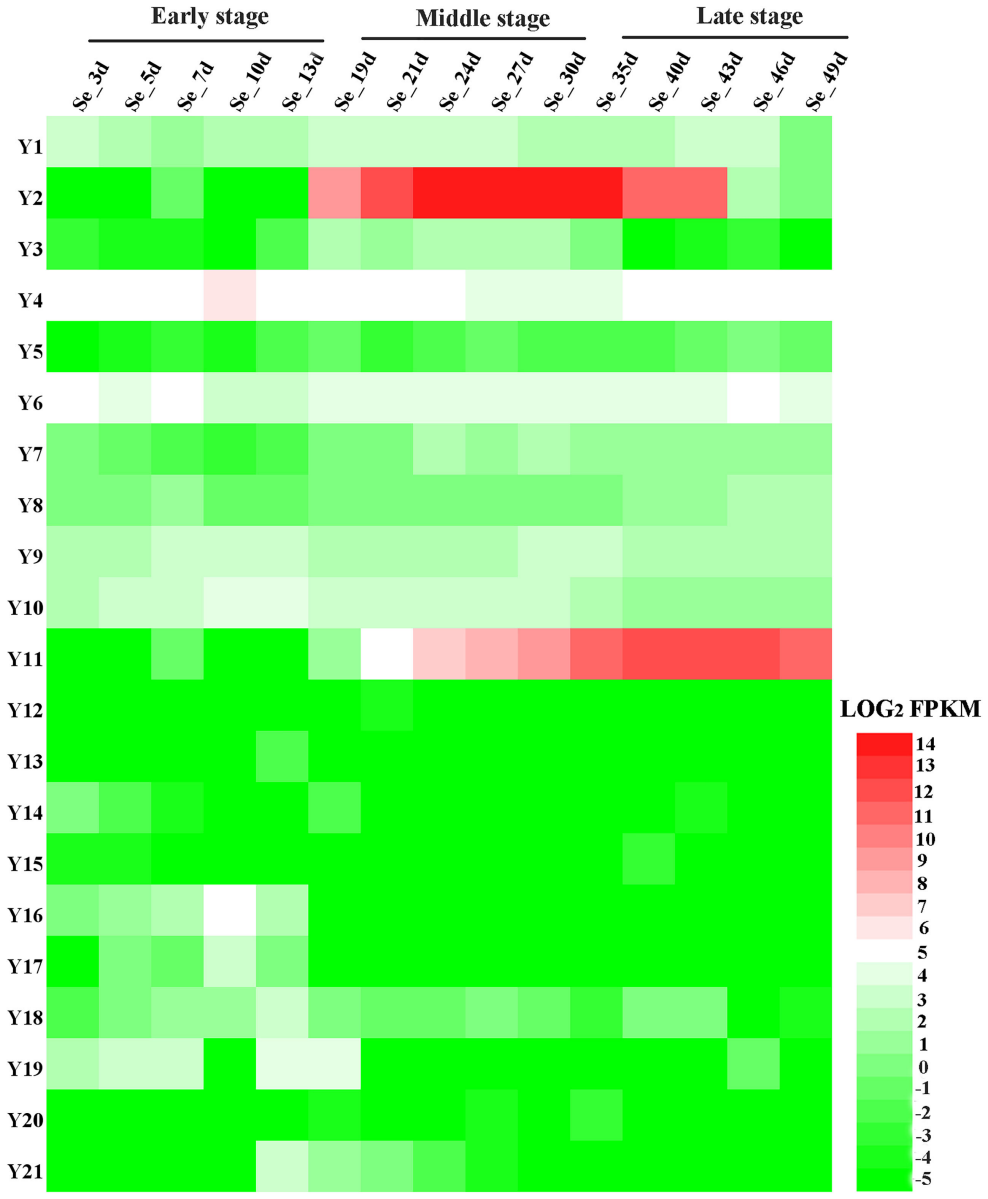
Expression patterns of 21 candidate genes in seeds at different developmental stages in *B. napus*. Se_-_3d, 5d, 7d, 10d, 13d, 19d, 21d, 24d, 27d, 30d, 35d, 40d, 43d, 46d, 49d represent seeds from *B. napus ZS11*, collected on the indicated number of days after pollination. The heatmap was drawn using Heatmap Illustrator (HemI) (Deng et al., 2014). The bar on the lower right corner represents LOG_2_FPKM; green and red represent low and high expression levels, respectively.

To explore the role of candidate genes in seed development, we investigated the expression profiles of all 21 genes in *B. napus ZS11* seeds at different developmental stages in the RNA-Seq dataset (BioProject ID PRJNA358784). The accuracy of the transcriptome sequencing has been verified in previous studies (Zhou et al., 2017; Di et al., 2018). 11 candidate genes (Y1-Y11) were expressed in seeds, but the levels often varied at different developmental stages (**Figure 4**; **Table S4**). For example, *BnaA03g48490D* (Y2) and *BnaA09g43150D* (Y11) were not expressed early in seed development, but were highly expressed in the middle and late stages. *BnaA01g22130D* (Y1), *BnaA03g50010D* (Y4), *BnaA03g52350D* (Y6), *BnaA03g53510D* (Y9), and *BnaA03g53860D* (Y10) showed little difference in expression over the course of seed development. *BnaA03g48800D* (Y3) was expressed in the middle stage of seed development, but was expressed at low levels in the early and late stages. *BnaA03g50730D* (Y5) exhibited relatively low expression across all stages. *BnaA03g52640D* (Y7) was expressed at higher levels in the middle and late stages than in the early stage, and the expression level of *BnaA03g52660D* (Y8) was higher in the late stage than in the early and middle stages of seed development. However, 5 candidate genes (Y12-Y15, Y20) were virtually unexpressed throughout seed development. Y16 and Y17 were only expressed early in seed development. The Y18 was expressed in small amounts throughout seed development, Y19 and Y21 genes were expressed during early and middle seed development. In general, the up-regulated candidate gene (Y1-Y11) in high-SOC accessions had higher expression levels than the down-regulated candidate gene (Y12-Y21) in the whole development process of seeds in *B. napus*.These results indicate that The up-regulated expression of 11 candidate genes may be more conducive to the formation of high-SOC. in *B. napus*.

## Discussion

*B. napus* is an important oil crops around the world. Increasing SOC is a major goal of *B. napus* breeding (Mollers and Schierholt, 2002; Vigeolas et al., 2007; Tan et al., 2011; Fu et al., 2017). In current study, we found that there is a significant positive correlation between SOC and economic yield and harvest index (**Table 2**). In *B. napus* breeding, enhancing the SOC may increase both economic yield and harvest index. Therefore, discovering genetic loci that control SOC, and uncovering their genetic mechanism are key to developing high-SOC varieties. Meanwhile, it is an effective way to identify candidate genes related to SOC and improve the SOC of *B. napus* by genetic engineering technology.

Although there have been many efforts to map QTLs controlling SOC, the loci discovered are different due to the use of different markers and populations. In current study, a new recombinant inbred population (RIL) containing 186 lines was constructed from a cross between ‘GH06’ and ‘ZY821’, two varieties with significant difference in SOC. A total of 26 QTLs were obtained, of which 13 are novel (**Table 3**). We were able to determine if they overlapped on the chromosomes by comparing the physical positions of QTLs obtained in this study with QTLs related to SOC obtained in previous studies using Darmor-bzh v4.1 reference genome of *Brassica napus* (Chalhoub et al., 2014). In this study, *2018-qOCA06-2* overlaps with the *RNSL-qOC-A6* obtained by Delourme et al (Delourme et al., 2006), *BLUP-qOCA03-1* overlaps with the *TN-qOC-A3-1*, and *2016-qOCA09-1*, *2016-qOCA09-2*, *2017-qOCA09-1*, *2017-qOCA09-2*, *2018-qOCA09-1*, *BLUP-qOCA09-1*, *BLUP-qOCA09-2* overlap with *qOC-A9-4-TN* obtained by Jiang et al (Jiang et al., 2014). *2016-qOCA09-2*, *2018-qOCA09-1*, *BLUP-qOCA09-1*, *BLUP-qOCA09-2* overlap with the *cqOC-A9-9*, *2018-qOCA09-2* overlaps with *cqOC-A9-10*, *2017-qOCC05-1* overlaps with cqOC-C5-7 and cqOC-C5-8 obtained in Chao’s study (Chao et al., 2017). Besides, the *BLUP-qOCA03-1* overlaps with the Bn-A03-p2948394, *2018-qOCA06-2* overlaps with Bn-A06-p16689717, and *2017-qOCA09-2* overlaps with Bn-A09-p32713083, *BLUP-qOCA09-1* overlaps with Bn-A09-p32713083 and Bn-A09-p32864411, and *2017-qOCC01-1* overlaps with Bn-scaff_17592_1-p654560 detected by Liu et al (Liu et al., 2016). The *2018-qOCA05-2* obtained in this study overlaps with the snp842906 obtained from Wang et al (Wang et al., 2018). The detected QTLs overlapped within our study and with other QTLs identified by others, indicating high reliability for further analysis.

In this study, 21 candidate genes (Y1-Y21) were screened related to SOC. However, 11 up-regulated candidate genes (Y1-Y11) in high-SOC accessions were considered to be candidates for the formation of high-SOC. Therefore, we will focus on these 11 candidate genes. Among all the 11 candidate genes (Y1-Y11) related to high-SOC formation, only Y1 (*BnaA01g22130D*), Y9 (*BnaA03g53510D*) and Y10 (*BnaA03g53860D*) were located in the novel QTL regions, and the remaining eight candidate genes were located in the overlapped QTLs detected previously. And there were seven candidate genes in the overlapped *BLUP-qOCA03-1* locus detected previously, which indicated that the *BLUP-qOCA03-1* may be a key locus associated with high SOC, which could provide a basis for marker-assisted breeding. And the homologous genes and functional descriptions of these genes in *Arabidopsis thaliana* were shown in **Table 4**. Among them, *BnaA01g22130D* (Y1), *BnaA03g52350D* (Y6) and *BnaA03g53860D* (Y10) are new proteins that have not been reported. The *Arabidopsis* homologs of other 8 candidate genes perform a variety of functions in growth, defense, and development (**Table 4**). Of these, we found *BnaA03g48490D* (Y2) is a putative seed storage albumin (*AtSESA3*). *BnaA03g48800D* (Y3) encodes an oxidoreductase required for proper development of the *Arabidopsis* vegetative shoot apex (Callos et al., 1994). *BnaA03g50010D* (Y4) is *AT4G30220* (*AtRUXF*) is a putative SmF component of Sm accessory ribonucleoprotein complex. *BnaA03g50730D* (Y5) encodes a putative (NAD+) aldehyde dehydrogenase. *BnaA03g52640D* (Y7) is LRR and NB-ARC domains-containing disease resistance protein. *BnaA03g52660D* (Y8) is a protein putatively involved in plastid to nucleus signaling (Lee et al., 2007). *BnaA03g53510D* (Y9) is a general transcription factor for RNA polymerase I (Imamura et al., 2008). And we find *BnaA09g43150D* (Y11), the Late embryogenesis-abundant (LEA) homolog, to be the most compelling candidate because of its direct role in embryogenesis. LEA proteins are considered to be a large and highly diverse family involved in normal plant growth, seed development and the abiotic stress response (Chen et al., 2019; Jin et al., 2019). To date, LEA proteins have been identified in *Arabidopsis* (Hundertmark and Hincha, 2008), rice (*Oryza sativa*) (Wang et al., 2007), apple (*Malus domestica*) (Liang et al., 2012), tomato (*Solanum lycopersicum*) (Cao and Li, 2015), black popular (*Populus trichocarpa*) (Lan et al., 2013), and sweet orange (*Citrus sinensis* L. Osb.) (Pedrosa et al., 2015). Furthermore, Liang et al. (2016) identified 108 *BnLEA* genes in the *B. napus* genome and classified them into eight families based on their conserved domains (Liang et al., 2016). A study showed that overexpression (OE) of different copies of the drought response genes *LEA3* (not Y11) enhanced both drought tolerance and oil content in *Brassica napus* and *Arabidopsis*, and seed size, seed weight and membrane stability were also improved in OE lines. In contrast, oil content and drought tolerance were decreased in the *AtLEA3* mutant (*atlea3*) of *Arabidopsis* and in BnLEA-RNAi *B. napus* RNAi lines (Liang et al., 2019). Therefore, we speculate that the candidate gene Y11 obtained in this study has an important function in improving seed oil content and abiotic resistance in *B. napus*.

To date, the gene function in *B. napus* of the 11 candidate genes (Y1-Y11) that contribute to the formation of high-SOC were highlighted in this study has not been reported. We hypothesize that the 11 novel genes identified in our study influence the SOC of *B. napus*. Therefore, in the following research, we plan to explore these genes further to provide a basis for the molecular breeding of high-SOC *B. napus*.

## Conclusions

We detected 26 QTLs associated with SOC in *B. napus*, that explained 3.69-18.47% of the phenotypic variation. Thirteen of these QTLs are reported here for the first time. And 1,713 candidate genes from the 26 QTLs mapping interval were obtained. Meanwhile, an analysis of DEGs between high- and low-SOC accessions revealed 21 candidate genes (Y1-Y21) related to SOC, and 11 candidate genes (Y1-Y11) contributing to the formation of high-SOC were highlighted. Current findings provide key information about the genetic loci associated with SOC, facilitating molecular breeding for higher SOC in *B. napus*.

## Data availability statement

The RNA-Seq datasets presented in this study can be found in online repositories. The names of the repository/repositories and accession number(s) can be found below: BIG Data Center under BioProject accession number PRJNA602979. And CQ46-30SM corresponds to CQ46-30ZS, CQ24-30SM corresponds to CQ24-30ZS, CQ52-30SM corresponds to CQ52-30ZS, CQ46-30SB corresponds to CQ46-30CS, CQ24-30SB corresponds to CQ24-30CS, CQ52-30SB corresponds to CQ52-30CS.

## Author contributions

JL and ZX conceived and designed the experiments. ZX, CZ, CQ, and LW performed the experiments. LZ, BY, and KL analyzed the data. ZX and JL wrote and revised the manuscript. All authors contributed to the article and approved the submitted version.
