# Supplementary material for: Identification of candidate genes regulating seed oil content by QTL mapping and transcriptome sequencing in Brassica napus
Source: Front Plant Sci. 2022 Dec 8;13:1067121. doi: 10.3389/fpls.2022.1067121 (PMC9779944; doi:10.3389/fpls.2022.1067121)
Supplement: Supplementary file 1 [file DataSheet_1.zip › Supplementary Tables/Supplementary_Material.docx]

Supplementary Material

**Supplementary Table 1 |** Seed oil content (SOC, % of seed weight) phenotypes in three environments (2016CQ-2018CQ).

**Supplementary Table 2 |** A list of all candidate genes in the QTL confidence interval.

**Supplementary Table 3 |** A list of primer sequences used in this study.

**Supplementary Table 4 |** Expression levels (FPKM) of 21 candidate genes in seeds at different developmental stages in *B. napus ZS11.*
